# Human West Nile Virus, France

**DOI:** 10.3201/eid1010.031021

**Published:** 2004-10

**Authors:** Pascal Del Giudice, Isabel Schuffenecker, Fréderic Vandenbos, Evelyne Counillon, Hervé Zeller

**Affiliations:** * Hôpital Bonnet, Fréjus, France;; † Institut Pasteur, Lyon, France

**Keywords:** letter, West Nile, France, southern France, encephalitis

**To the Editor:** West Nile virus (WNV) is a mosquito-transmitted flavivirus, widely distributed in Africa, the Middle East, Asia, and southern Europe. Since the 1990s, its geographic distribution has expanded and caused epidemics of meningoencephalitis (*1*). Recently introduced into the United States, it expanded rapidly from New York throughout the country and caused illness in 9,862 human patients in 2003 (*2*). In France, the first reported WNV outbreak that affected horses and humans occurred during the summer of 1962 in the Camargue region (*1*). After 1965, no human or equine WNV infections were reported until September 2000, when a large outbreak of equine encephalitis occurred in France (*3*). No human cases were reported at that time. In September 2003, a human living in Fréjus (Département du Var, southeastern France) was diagnosed with acute WNV infection in Nice University Hospital. At the same time, an equine case was diagnosed 20 km from the patient’s home; consequently, public health authorities initiated a retrospective study of patients hospitalized in the French Mediterranean region in which viral meningoencephalitis was suspected. We report four human cases from Fréjus Hospital.

Twenty patients who had been hospitalized at some time from August 1 to October 15, 2003, for febrile meningitis, encephalitis, or polyradiculoneuritis were screened. Four patients in whom cerebrospinal fluid (CSF) analysis indicated a viral cause were included. In addition, serum samples from two patients who had experienced flulike symptoms with exanthema during the same period were tested further. Serologic diagnosis of acute WNV infection was based on immunoglobulin (Ig) M-capture and direct IgG enzyme-linked immunosorbent assay followed by 80% plaque reduction neutralization titer (PRNT_80_) by using the France 2000 WNV strain (*3*).

Patient 1, 46 years old, and patient 2, 25 years old, had a flulike syndrome with maculopapular exanthema; WNV seroconversion was seen on a pair of sera collected on days 3 and 16 for patient 1, and days 3 and 12 for patient 2, after onset of fever. Patients 3 and 4 had meningoencephalitis with maculopapular exanthema. In patient 3, a fourfold increase in WNV neutralizing antibodies was seen in serum samples on 2 consecutive days (days 3 and 15 after onset of fever). In patient 4, WNV IgM antibodies were detected in CSF (day 4 after onset of fever), and neutralizing antibodies (titer = 160) were reported in a serum specimen on day 75. Attempts to detect WNV RNA by reverse transcription–polymerase chain reaction, or to isolate the virus from serum specimens in patients 1 and 2 and CSF in patient 4, were negative because of the low level and short duration of WNV viremia (*4*). All patients recovered.

On the basis of serologic results, we describe the first human clinical WNV infections in France since 1964 (*5*). The four patients lived in the same city, had not traveled, and had an onset of their illness during the last week of August 2003. Of note, four clinical infections were identified, but many more WNV subclinical and asymptomatic infections likely occurred simultaneously.

After the reemergence of WNV in horses in the Camargue region in 2000, surveillance on sentinel birds (ducks and chickens) showed a low circulation of WNV in 2001 and 2002 in this area. Meanwhile, no clinical human or equine cases were detected. During the summer of 2003, WNV reemerged in humans 200 km east of Camargue, in the Département du Var, along the Mediterranean coast. A study conducted on French blood donors from September to November 2000 showed low titers of WNV neutralizing antibodies in two donors originating from the Département du Var (*6*). However, to date, no clinical human cases have been reported in this area.

WNV must be considered as a causative agent of meningitis, encephalitis, and polyradiculoneuritis during summer and early fall in southern France. Given the capacity of WNV to cause large outbreaks, the surveillance will be extended to the entire Mediterranean coastal area.
